# Supplementary material for: Patterns of Intron Gain and Loss in Fungi
Source: PLoS Biol. 2004 Nov 30;2(12):e422. doi: 10.1371/journal.pbio.0020422 (PMC532390; doi:10.1371/journal.pbio.0020422)
Supplement: Table S1 — Also available at http://genes.mit.edu/NielsenEtAl/. (4.3 MB ZIP). [file pbio.0020422.st001.zip › NielsenEtAl/html/167.html]

AN2057.1.NCU06469.1.MG06378.1.FG07257.1


```
 CLUSTAL W (1.82) Multiple Sequence Alignments - Introns Inserted


Sequence 1: MG06378.1	177 aa
Sequence 2: FG07257.1	180 aa
Sequence 3: NCU06469.1	176 aa
Sequence 4: AN2057.1	184 aa
Alignment Length: 186 aa
Number Identitical Residues: 92 aa
Alignment Score (without introns) 4008


MG06378.1 	MSVSCRYAAQCCSRQLRVSPSVRVSG--VAVQRIARRHNSTEAA----SPKITTIVDQIS
NCU06469.1	MSLSVRFAAQCAARQLRAS--TRASS----SLLVQKRFESTAAPAA--NPKIAAIVDQIS
FG07257.1 	MAMSCRYAAQSCARQLRSARTARAPAQLVRIPATSRRWNSTEAAPV--NPKIEAIVDQIS
AN2057.1  	MSITSQAAARCCRQLVRPSASLRLSA--TYQQTISRRWQSTEAPAAPANPKITQIVDQIS
          	*::: : **:.. : :* : : * ..         :* :** *.. .:.***  ******

MG06378.1 	QLTLLETAELVSSLK0SRLNIPDLPVGGFAAAAPAAAP--AAA-EEADEPAPAAAAKTVF
NCU06469.1	TLTLLETSELVSSLK0SRLNIPDLPVGGFAAAAPAAAP--AAAPAEEEEAAPAAAEKTLF
FG07257.1 	QLTLLETADLVSSLK0NKLNIPDMPIGGFAAA-PAAAP--AAV-EEAEEAAPAAAEKTLF
AN2057.1  	QLNLLETADLVASLK0SRLNIPDLPVGGFAMAPGAGAPGAAAAPAEEEEAAPPAQEKTLF
          	 *.****::**:*** .:*****:*:**** *. *.**.:**.. * :*.**.*  **:*

MG06378.1 	TLKLMSFDAGAKAKIIKEVKGLLGLSLVDSKKFVESAPKVMKESVPKEDAEKIIATMKEL
NCU06469.1	TLKLEKFDAAAKPKVIKEVKNLLGLSLVESKKFVESAPKVMKESVPKEDAEKIVAAMKEL
FG07257.1 	TLKLQGFDAPSKAKVIKEVKNMLGLSLVDSKKFVESAPKMMKENVPKEDAEKIINTMKEL
AN2057.1  	NLKLESVDAAQKAKVIKEVKTLLGLSLVDSKKFVESVPKVLKESVPKEEAEKLIETLKAV
          	.***  .**  *.*:***** :******:*******.**::**.****:***:: ::* :

MG06378.1 	GAVVTME
NCU06469.1	GATVTMD
FG07257.1 	GATVVME
AN2057.1  	GAKAVME
          	** ..*:
```
